# Complications and Mortality Rate of Vagus Nerve Stimulation for Drug-Resistant Epilepsy

**DOI:** 10.7759/cureus.63842

**Published:** 2024-07-04

**Authors:** Yitao Ma, Nicholas Lehman, Robert Crutcher, William Young, David Horvat

**Affiliations:** 1 Neurology, Walter Reed National Military Medical Center, Bethesda, USA; 2 Pediatric Medicine, Walter Reed National Military Medical Center, Bethesda, USA; 3 Neurology, Uniformed Services University of the Health Sciences (USUHS), Bethesda, USA

**Keywords:** drug-resistant epilepsy, sudep (sudden unexplained death in epilepsy), av block, sleep apnea, complications, vagus nerve stimulation

## Abstract

Objective: The goal of this study is to evaluate the complications and mortality associated with vagus nerve stimulation (VNS).

Methods: We retrospectively reviewed medical records of patients who underwent VNS implantation for the treatment of drug-resistant epilepsy (DRE) between 2000 and 2023. The mean follow-up time was 10.6 years, ranging from three months to 22 years.

Results: In total, 55 adult and pediatric patients received VNS therapy with 117 procedures performed over 23 years. The most common early complications were hoarseness and cough which were reported in eight adult patients (6.8%). Four children with intellectual disability (ID) had infection (3.4%), eight patients had lead breakage (6.8%), and two had device migration (1.7%). Four of all patients (7.3%) demonstrated late complications due to chronic nerve stimulation including vocal cord dysfunction, late-onset severe AV block, and obstructive sleep apnea (OSA). Three patients (5.5%) had VNS deactivated permanently due to complications and/or lack of efficacy. Two patients died from probable sudden unexpected death in epilepsy (SUDEP) with an incidence of 3.4/1000 person-years.

Conclusions: VNS therapy is safe over long-term follow-up but not without risks. Most post-operative complications are minor and transient for adults. Children with ID tend to have infection and device migration. Late-onset cardiac complications and OSA can develop in some patients during VNS therapy and should not be overlooked. The SUDEP rate may decrease with VNS therapy over time.

## Introduction

Vagus nerve stimulation (VNS) is an alternative treatment option for individuals with drug-resistant epilepsy (DRE) who are not good candidates for surgical resection. VNS was approved by the U.S. Food and Drug Administration (FDA) for the treatment of DRE in 1997. The approval was extended to children aged 4 years or older in 2017. VNS is the most cost-effective and the most frequently used neuromodulation [1,2]. Approximately 45-65% of patients achieved at least 50% reduction in seizure frequency with VNS therapy [3].

The VNS system includes a pulse generator implanted in the chest, which is connected to a bipolar lead that wraps around the left vagus nerve within the carotid sheath [4]. Due to the presumed risk of stimulation-induced cardiac arrhythmia, the left side is the preferred side for stimulation [5]. The exact mechanism of action of VNS therapy for DRE is unknown. The VNS system, by delivering acute and chronic stimulation to the vagus nerve, is believed to modulate neural circuits involved in seizure generation and propagation. Chronic stimulation may modulate GABAergic function and decrease neuroinflammation [4,6,7].

Benefits of VNS treatment include a better tolerability profile than many anti-seizure medications (ASMs). VNS is a lifelong treatment for many patients with DRE. Current long-term safety data showed that VNS therapy is safe albeit with certain risks [8]. The complications of VNS therapy can be classified as early complications (related to surgery) and late complications (related to chronic nerve stimulation) [9]. Common complications related to surgery include infection, hematoma, lead breakage, or symptoms related to traction of the inferior recurrent laryngeal nerve including hoarseness, dyspnea, and dysphagia. Late complications may show up as delayed bradyarrhythmia, obstructive sleep apnea (OSA), and vocal cord dysfunction. In this study, we included both early and late complications related to VNS therapy over the past 23-year period.

## Materials and methods

The study was approved by the institutional review board. We performed a retrospective chart review, gathering clinical, radiographic and electrophysiologic data on consecutive patients with DRE who were treated with VNS at Walter Reed National Military Medical Center (WRNMMC) from January 1, 2000, to December 31, 2023. All variables including demographic data of patients, the occurrence of surgical complications, VNS settings and VNS outcome were documented in Microsoft Excel spreadsheets.

All surgeries including primary generator implantation, pulse generator replacement, revision, and lead replacement were performed by neurosurgeons or otolaryngologists of WRNMMC and took place under general anesthesia. The surgical field was disinfected with chlorhexidine. The procedure was performed under strict sterile conditions. The surgical procedure was the same for all patients, both children and adults. All patients underwent continuous monitoring for five hours after the procedure and were discharged on the same day if no complications were noted. The device was normally programmed to start stimulation two weeks after surgery.

In this study, a complication is defined as an unintended effect caused by a surgical procedure and/or related to VNS stimulation. Early complications are related to surgery, and late complications are related to chronic stimulation.

Patient characteristics were analyzed and summarized using descriptive statistics. Continuous variables were presented as mean with standard deviation (SD), and categorical variables were presented as a number with a percentage. The descriptive nature of this study and the small sample size did not necessitate analysis for statistical significance.

## Results

Demographic data

In total, 55 patients with 117 VNS procedures performed from 2000 to 2023 were included in the study. Fifty-three procedures (45.3%) were performed on children. At the time of the study, 43 adults (78.2%) with a mean age of 37.0 and 12 children (21.8%) with a mean age of 12.8 had VNS implantation. The mean age at primary implantation was 26.6 years for adults and 8.7 years for children. The duration of epilepsy at the time of primary implantation was 13.0 years for adults and 6.1 years for children. Duration of VNS therapy averaged 10.6 years ranging from three months to 22 years. 22 patients (40%) had lesional epilepsy, and four patients (7.3%) were diagnosed with genetic generalized epilepsy (GGE). Detailed demographic data are listed in Table 1. The procedures consisted of primary implantation of the VNS system (n = 55), replacement of the VNS pulse generator (n = 46), replacement of the leads (n = 2), and incomplete VNS removal surgery (n = 6), or any other VNS related surgery (n=8). VNS surgery-related complications were reported in 24.8% of all procedures. Four patients (7.3%) suffered from late complications from chronic stimulation. 

**Table 1:** Patient characteristics

	Total	Adults	Children
No.	55	43	12
No. of males (%)	30 (54.5)	22 (51.2)	8 (66.7)
Mean age, yrs (SD)	31.7 (16.9)	37.0 (15.3)	12.8 (2.3)
Mean age of seizure onset, yrs (SD)	11.2 (13.6)	13.7 (14.5)	2.6 (2.4)
Mean age at primary implantation, yrs (SD)	22.7 (16.1)	26.6 (16.0)	8.7 (4.8)
Duration of Epilepsy at implantation, yrs (SD)	11.5 (9.0)	13.0 (9.5)	6.1 (3.9)
Duration of VNS therapy, yrs (SD)	10.6 (6.2)	11.9 (6.2)	5.8 (3.3)
No. of lesional epilepsy (%)	22 (40)	15 (34.9)	7 (58.3)
No. of genetic generalized epilepsy (%)	4 (7.3)	3 (7.0)	1 (8.3)

Adverse events and complications

Post-operative infection of generator pockets was reported in four children (3.4%) with intellectual disability (ID). One patient had the VNS generator permanently removed after infection. Two infections occurred after primary implantation and two after pulse generator replacement. All patients received oral antibiotics as the first line of treatment. One child had a peripherally inserted central catheter (PICC) line placed and received intravenous antibiotics for weeks. All were treated with surgical removal of the stimulator and proximal part of the leads. Two patients eventually had right VNS placement due to refractory seizures without further signs of infection.

Post-operative dysphagia was seen in three adult patients (2.6%). Two adult patients developed symptoms of mild dysarthria, dysphagia, and cough after primary implantation which lasted for weeks and then resolved. The other patient suffered persistent dysphagia and eventually chose to have her VNS deactivated. 

Five adult patients (4.3%) complained of post-operative voice alteration or hoarseness which improved over time. Three adult patients (2.6%) suffered a cough that was more intense during interrogation. All these patients remained on low output current setting due to poor tolerance. Two adult patients (1.7%) suffered from transient headaches after device implantation that resolved after four months. Lead breakage or malfunction was reported in two adult patients (3.1%) and six pediatric patients (11.3%). Two pediatric patients (1.7%) with ID were found to have device extrusion or migration. All resulted in the replacement of the generator. Three of all patients (5.5%) had VNS deactivated permanently due to complications and/or lack of efficacy. All adverse events and complications are summarized in Table 2.

**Table 2:** Early complications associated with VNS therapy VNS: Vagus nerve stimulation

	Total No. (n=117)	Adults No. (n=64)	Children No. (n=53)
Hoarseness	5 (4.3%)	5 (7.8%)	0 (0.0%)
Cough/dyspnea	3 (2.6%)	3 (4.7%)	0 (0.0%)
Dysphagia	3 (2.6%)	3 (4.7%)	0 (0.0%)
Paresthesia	2 (1.7%)	2 (3.1%)	0 (0.0%)
Headaches	2 (1.7%)	2 (3.1%)	0 (0.0%)
Infection	4 (3.4%)	0 (0.0%)	4 (7.5%)
Lead breakage	8 (6.8%)	2 (3.1%)	6 (11.3%)
Device migration	2 (1.7%)	0 (0.0%)	2 (3.8%)

Four of all patients (7.3%) demonstrated late complications from chronic stimulation. One adult patient and one pediatric patient (3.6%) developed vocal cord paralysis confirmed by laryngoscopy three years after starting VNS therapy. They both also developed OSA.

One patient (1.8%) developed severe atrioventricular (AV) block three years after VNS implantation. She had no history of heart disease. There was no reported intraoperative or perioperative bradycardia. She was doing well initially but developed new syncope episodes with falls which were later confirmed to be due to third-degree AV block. She received a right-sided dual chamber pacemaker implantation to complement her left-sided VNS and subsequently experienced no further syncope events.

Two adult patients and one pediatric patient (5.5%) were diagnosed with OSA after initiation of VNS therapy. The male adult patient had a body mass index (BMI) of 25. Polysomnography (PSG) confirmed diagnosis of mild positional OSA with a supine apnea-hypopnea index (AHI) of 11, requiring automatic positive airway pressure treatment. His supine events only occurred in the middle part of the study just before and after stopping the VNS. The PSG record of the female adult patient is not retrievable. Available records indicated she was diagnosed with OSA and was treated with CPAP. The pediatric patient was diagnosed with severe OSA at age eight. All three patients’ VNS was set on high intensity when OSA was diagnosed. All of them were able to continue VNS therapy with PAP treatment. OSA resolved after uvulopalatopharyngoplasty (UPPP) in the pediatric patient. The detailed information is listed in Table 3.

**Table 3:** Characteristics of patients with OSA AHI: apnea-hypopnea index; RDI: respiratory disturbance index; SaO_2_: arterial oxygen saturation; OSA: obstructive sleep apnea.

Patient	1	2	3
Age (yrs), Gender	68, M	62, F	20, F
Age at VNS implantation (yrs)	65	50	2
Age at diagnosis of OSA (yrs)	67	54	8
BMI	25	26	20
Sleep study results	mild positional OSA	OSA	severe OSA
AHI	supine/non-supine 11/1.7	-	40.1
RDI	supine/non-supine 11/3	-	51
min % SaO2	84	-	90
VNS setting at diagnosis of OSA			
Output current (mA)	1.5	2.5	2
Duty cycle %	35	25	49
Magnet current (mA)	1.75	2.75	2.25

Mortality

Four patients have died over the 23 years. One patient died from a generalized tonic-clonic seizure that led to a fall which resulted in large subdural hemorrhage. Two patients died from probable sudden unexpected death in epilepsy (SUDEP). One of them died from probable SUDEP within one year after VNS implantation. The other patient died 3.7 years after VNS implantation. There was no reported SUDEP from patients who had VNS therapy for longer than four years. The incidence of SUDEP is 3.4 per 1000 person-years. One died from medical complications. All had recent VNS interrogation and were without signs of device malfunction.

## Discussion

VNS was the first neuromodulation approved as an adjunctive treatment for DRE. Many studies have been done to investigate its safety and efficacy [3]. The reported complication rate from surgery or chronic stimulation ranges from 0-27.3% [8,10,11]. In our study, the rate of early complications from surgery is 24.8%. Four patients (7.3%) were noted to have late complications from chronic stimulation. Almost all transient complications were reported by adult patients. While infection, device migration, and 75% of the lead breakage cases were seen in pediatric patients, the majority of these patients suffered from epileptic encephalopathy with ID and behavioral changes which may contribute to abnormal manipulation of the device. Our results are consistent with previous studies which indicated ID is a risk factor for infection and device malfunction [12].

One patient developed late-onset severe AV block three years after primary VNS implantation. The etiology of her late-onset bradyarrhythmia is likely multifactorial. The vagus nerve is a parasympathetic nerve that can regulate the autonomic tone of many internal organs including the heart. Cardiac branches from the left vagus nerve modulate the activity of the AV node while the branches from the right vagus nerve can affect the sinoatrial (SA) node. This raised concern that chronic VNS can affect heart conduction and rhythm. There were rare reports of intra-operative and post-operative asystole or severe bradycardia [13-15]. Additionally, several case reports described late-onset bradycardia or complete AV block occurring years after the device implantation, resulting in the termination of VNS therapy for all affected patients [5,16-18]. Considering the large number of VNS devices implanted, the risk of cardiac complications is very low. The VNS leads are advised to be attached to the trunk of the vagus nerve which is devoid of any cardiac branches. Anatomic variations of the vagus nerve or its innervation to the SA and AV nodes may be the underlying mechanisms of cardiac complications. Changes in the autonomic functions in the hypothalamus and insular cortex from chronic stimulation may play a role in late-onset cardiac complications [5]. Many ASMs are known to affect heart conduction and rhythm. The patient in our study was on two sodium channel blockers including lacosamide and rufinamide which may also have contributed to her cardiac arrhythmia. In 2021, the FDA issued a warning of cardiac complications from the sodium channel-blocking properties of lamotrigine [19]. It is unclear if there is a synergistic interaction between VNS and sodium channel blockers that can potentially increase the risk of adverse cardiac effects. Since sodium channel blockers are commonly used ASMs, it is important to monitor these patients with periodic EKG to screen for this severe complication. Urgent epilepsy monitoring unit admission is recommended to characterize new syncope or near syncope events. Patients who are responders to VNS can continue the treatment with cardiac pacemaker placement.

Three patients were diagnosed with OSA after starting VNS therapy. VNS was set at high intensity for all three patients. It appears the occurrence of OSA is dose-dependent. Two of them also had concurrent vocal cord dysfunction confirmed by laryngoscopy. All continued VNS therapy with PAP treatment. The pediatric patient was able to wean off BiPAP after UPPP. The prevalence of OSA in VNS patients is unknown. Limited studies have found an increased incidence and severity of OSA after VNS implantation and that higher VNS intensities were strongly related to increased AHI [20-23]. Zambrelli et al. proposed that OSA may be related to stimulation-induced left vocal cord adduction and can be controlled by adjusting VNS parameters [21]. However, Gschliesser et al. and Dye et al. found that the AHI and oxygen desaturation index were not affected by different VNS modes [22,24]. The relationship between VNS and OSA needs to be further elucidated. The presence of OSA may exacerbate epilepsy [25], while treatment of OSA can improve seizure control and the quality of life for patients with DRE [26]. It is therefore important to recognize OSA and start treatment accordingly.

The risk of premature death is higher in patients with DRE. A significant proportion of these deaths are epilepsy-related, including SUDEP. Previous studies indicated that VNS therapy will not affect the rate of SUDEP in patients with DRE [27]. However, a more recent study showed that VNS therapy could significantly decrease the rate of SUDEP over time [28]. The incidence of SUDEP is 3.4 per 1000 person-years which is lower than the reported 5.6 per 1000 person-years for patients with chronic refractory epilepsy [29] and 9.3 incidence of SUDEP for patients who are epilepsy surgery candidates [30]. Our study is limited by a small sample size, but we still can detect a trend of decreased incidence of SUDEP with longer VNS therapy since the two patients had VNS for less than four years.

The strength of the study is the long-term follow-up of patients with VNS therapy. The longest follow-up time was 22 years. Our study is limited by its retrospective design and relatively small sample size. There are missing data due to the transition of the medical record system. Pediatric patients with ID may not complain of certain symptoms as adult patients. PSG was not routinely performed in all patients before and during VNS therapy. Patients with mild symptoms of OSA may not seek medical attention. The true incidence of OSA with chronic VNS therapy is unknown. Further studies are needed to evaluate how epilepsy, ASMs, and VNS affect cardiac rhythm, sleep, and mortality of patients with DRE.

## Conclusions

VNS therapy is relatively safe but also involves some risks. The most common complications for adults are minor and transient. Children are prone to postsurgical infection, lead breakage, and generator migration which require surgical revision. AV block can be seen years after VNS implantation. Urgent EKG monitoring is recommended for patients who develop new syncope or pre-syncope events. OSA can develop with VNS therapy and may be dose-dependent. Further prospective studies are needed to clarify the relationship between VNS and OSA. The risk of SUDEP may decrease with VNS therapy over time. Although being considered a palliative care procedure, VNS therapy should be offered to patients who are not candidates for surgical resection.
